# *Ecklonia cava* Attenuates PM_2.5_-Induced Cognitive Decline through Mitochondrial Activation and Anti-Inflammatory Effect

**DOI:** 10.3390/md19030131

**Published:** 2021-02-27

**Authors:** Seon Kyeong Park, Jin Yong Kang, Jong Min Kim, Hyun-Jin Kim, Ho Jin Heo

**Affiliations:** Division of Applied Life Science (BK21), Institute of Agriculture and Life Science, Gyeongsang National University, Jinju 52828, Korea; skpark200469@gnu.ac.kr (S.K.P.); kangjy2132@gnu.ac.kr (J.Y.K.); myrock201@gnu.ac.kr (J.M.K.); hyunjkim@gnu.ac.kr (H.-J.K.)

**Keywords:** *Ecklonia cava*, PM_2.5_, antioxidant, anti-inflammation, mitochondrial function, cognition

## Abstract

To evaluate the effects of *Ecklonia cava* (*E. cava*) on ambient-pollution-induced neurotoxicity, we used a mouse model exposed to particulate matter smaller than 2.5 µm in aerodynamic diameter (PM_2.5_). The intake of water extract from *E. cava* (WEE) effectively prevented the learning and memory decline. After a behavioral test, the toll-like receptor (TLR)-4-initiated inflammatory response was confirmed by PM_2.5_ exposure in the lung and brain tissues, and the WEE was regulated through the inhibition of nuclear factor-kappa B (NF-κB)/inflammasome formation signaling pathway and pro-inflammatory cytokines (IL-6 and IFN-γ). The WEE also effectively improved the PM_2.5_-induced oxidative damage of the lungs and brain through the inhibition of malondialdehyde (MDA) production and the activation of mitochondrial activity (mitochondrial ROS content, mitochondria membrane potential (MMP), adenosine triphosphate (ATP) content, and mitochondria-mediated apoptotic molecules). In particular, the WEE regulated the cognition-related proteins (a decreased amyloid precursor protein (APP) and p-Tau, and an increased brain-derived neurotrophic factor (BDNF)) associated with PM_2.5_-induced cognitive dysfunction. Additionally, the WEE prevented the inactivation of acetylcholine (ACh) synthesis and release as a neurotransmitter by regulating the acetylcholinesterase (AChE) activity, choline acetyltransferase (ChAT), and ACh receptor (AChR)-α3 in the brain tissue. The bioactive compounds of the WEE were detected as the polysaccharide (average Mw; 160.13 kDa) and phenolic compounds including 2′-phloroeckol.

## 1. Introduction

Air pollution is a current and growing global problem [1]. Fine dust (particulate matter; PM_2.5_) smaller than 2.5 µm in aerodynamic diameter can cause various health hazards and other systemic dysfunctions. PM_2.5_ is derived from various origins and is composed of primary emissions from anthropogenic and natural sources and secondary formation by chemical reactions (e.g., NO^3−^, SO^4−^, NH^4−^, polyaromatic hydrocarbon (PAHs), quinone et al.) [1]. Absorbed PM_2.5_ is not easily removed from the body and has immediate effects such as oxidative stress and inflammatory-mediated reaction. PM_2.5_ passes through the filtration of the nose hair and reaches the lung tissue at the end of the respiratory tract with central airways [2]. The accumulated PM_2.5_ directly causes pulmonary dysfunction by the systemic oxidative damage and an inflammatory reaction [1]. In lung tissue, PM_2.5_ stimulated oxidative stress, inflammation, and DNA damage lead to serious lung damage and airway diseases (e.g., allergic airway inflammation, asthma, and chronic obstructive pulmonary disease) [3].

Inflammation from PM_2.5_ in the lungs leads to systemic inflammation through the circulatory system [4]. Additionally, pulmonary damage by air pollution influences CNS disease and causes damage by producing the circulating cytokine response through the lung-brain axis [5]. The circulating factors (e.g., serum C-C motif chemokine ligand (CCL)2, CCL11, TNF-α, IL-6, and IL-1β) are detected by microglia in the brain tissue, and microglia triggers neuronal damage, neuroinflammatory response, and CNS diseases including Alzheimer’s disease (AD), Parkinson’s disease (PD), and autism [5,6]. Moreover, high levels of PM_2.5_ can reach the brain directly without having to go through the brain–blood barrier (B-B-B) through olfactory epithelial cells. The magnetic particles are deposited and cause neuroinflammation and cognitive impairment by pro-inflammatory cytokines (IL-1, TNF-α, and IFN-γ) [7]. Neuroinflammation by PM exposure causes amyloid-beta peptide production and tau hyperphosphorylation, thereby causing cognitive dysfunctions such as AD [8,9,10]. For this reason, it is necessary to study substances that can prevent environmental pollution (PM_2.5_)-induced cognitive decline.

*E. cava* is a major edible brown seaweed that grows along the coast of South Korea and is composed of unique physiological bioactive substances such as phlorotannins, sulphated polysaccharides, peptides, carotenoids, and fucoidans [11,12,13]. The physiological activity of *E. cava* is still unclear, and it has been reported that their biological mechanisms have not been elucidated. Recently, as mentioned in some reports, phlorotannins (e.g., eckol, bieckol, dieckol) among their bioactive compounds are considered the most important compound in the aspect of antioxidant activity [11]. In addition, fucoidans have been reported as potential sources for anti-coagulative, anti-obesity, and anti-inflammatory effects [14]. Therefore, we tried to evaluate the health benefits of *E. cava* on PM_2.5_-exposed mice and have confirmed its potential as a functional ingredient.

## 2. Results

### 2.1. Behavioral Test

**Figure 1:**
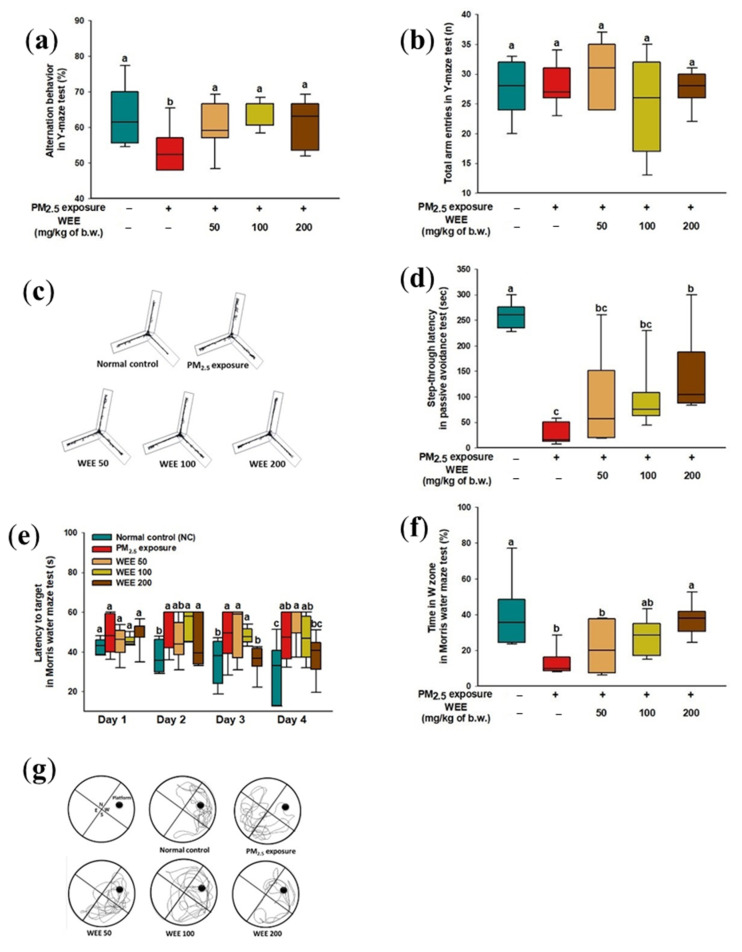
Effect of water extract from *Ecklonia cava* (WEE) on fine dust (PM_2.5_)-induced learning and memory impairment mice. Alternation behavior (**a**), total arm entries (**b**), and the path tracing of each group (**c**) in the Y-maze test. Step-through latency time in passive avoidance test (**d**). Escape latency in the hidden-platform training trial (**e**), time in W zone for probe trial (**f**), and path tracing in the probe trial (**g**) in the Morris water maze test. Bars in box plot are colored by group (green-colored bars; normal control (NC), red-colored bars; PM_2.5_ exposure (PM_2.5_), yellow-colored bars; WEE 50, light green-colored bars; WEE 100, brown-colored bars; WEE 200). The results are shown with a mean of ± SD (*n* = 9), and they were considered statistically significant at *p* < 0.05. Different small letters ^(a–c)^ indicate a statistical difference.

The results of the Y-maze test were shown to impair working spatial memory in alternation behavior by PM_2.5_ exposure (53.60%) compared with the normal control (NC) (63.02%) in Figure 1a. On the other hand, the administration of the water extract from *E. cava* (WEE) (50: 60.73%; 100: 64.42%; 200: 61.43%) effectively prevented working spatial memory impairment. The number of total arm entries showed a similar result for all groups (average 27.00) (Figure 1b) The black lines indicate the movement of mice in Figure 1c, and the NC showed a similar path tracing in each arm. On the other hand, the PM_2.5_-exposed group tended to lean toward one arm and distracted movement within the arm, and similar movements to the NC group were found in the WEE groups.

To evaluate the short-term learning and memory function, a passive avoidance test was conducted. The PM_2.5_-exposed mice (26.83 s, about an 89.68% decrease) showed significant short-term learning and memory impairment compared to the NC group (260 s) (Figure 1d), and the WEE groups (WEE 50: 94.17 s, WEE 100: 99.33 s, WEE 200: 144.67 s) prevented the short-term learning and memory impairment.

To evaluate the long-term learning and memory functions, the Morris water maze test was conducted. For 4 days in the hidden platform training session (Figure 1e), all mice tended to decrease the amount of time searching for the hidden platform. On the fourth day of the training session, the PM_2.5_-exposed group (45.95 s) indicated a higher escape latency time than the NC group (32.75 s), while the WEE 200 group (38.35 s) had a significantly decreased escape time. In the probe test (Figure 1f,g), the PM_2.5_-exposed group showed decreased learning and memory for the platform (13.60 s, about a 66.84% decrease) compared with the NC group (41.01 s). The WEE 200 group showed a similar range of learning and memory for the platform (37.54 s) compared with the NC group. As shown in Figure 1g, it was confirmed that the mice of the NC and WEE groups showed more movement in the W zone compared to the PM_2.5_-exposed group.

### 2.2. Anti-Inflammatory Effect

**Figure 2:**
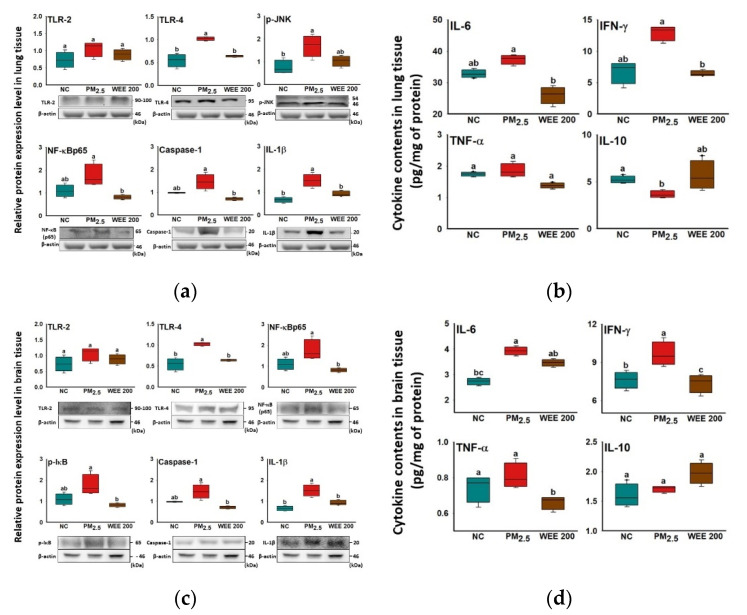
Anti-inflammatory effect of water extract from *Ecklonia cava* (WEE) on fine dust (PM_2.5_)-induced inflammation in lung and brain tissue. Band images of Western blot analysis and the expression level of inflammation-mediated molecules (**a**) and cytokine content (**b**) in lung tissue. Band images of Western blot analysis and the expression level of inflammation-mediated molecules (**c**) and cytokine content (**d**) in brain tissue. Bars in box plot are colored by group (green-colored bars; normal control (NC), red-colored bars; PM_2.5_ exposure (PM_2.5_), brown-colored bars; WEE 200). The results are shown with a mean of ± SD (*n* = 5), and they were considered statistically significant at *p* < 0.05. Different small letters ^(a–c)^ indicate a statistical difference.

TLR-4 was expressed by PM_2.5_ exposure in lung tissue, while statistical differences were not found in TLR-2 (Figure 2a). The TLR-4-initiated inflammatory response led to *p*-JNK expression and the activation of the NF-κB signaling pathway by increasing the NF-κB and *p*-IκB expression in the PM_2.5_-exposed group. As a result of the *p*-JNK expression and NF-κB signaling pathway, inflammasome formation was induced and activated caspase-1, and IL-1β expression increased in the PM_2.5_-exposed group. In addition, the activation of the NF-κB signaling pathway caused the production of pro-inflammatory cytokines. Among the pro-inflammatory cytokines, IFN-γ showed a significant increase (Figure 2b). On the other hand, anti-inflammatory cytokine (IL-10) was inhibited in the PM_2.5_-exposed group. The administration of the WEE 200 effectively blocked the PM_2.5_-induced inflammatory reaction by inhibiting the NF-κB signaling pathway and inflammasome formation. As a result, the pro-inflammatory cytokine (IL-6 and IFN-γ) production was inhibited in the WEE 200 group compared with the PM_2.5_-exposed group.

In brain tissue, the results indicated that the brain inflammation was also initiated by TLR-4 expression (Figure 2c). The TLR-4 expression led to the activation of the NF-κB pathway and inflammasome formation, and it activated the IL-1β expression, which increased in the PM_2.5_-exposed group. In addition, the activation of the NF-κB signaling pathway caused the production of pro-inflammatory cytokines (IL-6 and IFN-γ), while the anti-inflammatory cytokine (IL-10) was not inhibited in the PM_2.5_-exposed group (Figure 2d). The administration of the WEE 200 effectively regulated the PM_2.5_-induced inflammatory reaction by inhibiting the NF-κB signaling molecules (NF-κB and *p*-IκB) and inflammasome formation (caspase-1 and IL-1β) compared with the PM_2.5_-exposed group. As a result, the pro-inflammatory cytokine (IFN-γ) by was effectively inhibited PM_2.5_-exposure in the WEE 200 group.

### 2.3. Inhibitory Effect of Lipid Peroxidation

**Figure 3:**
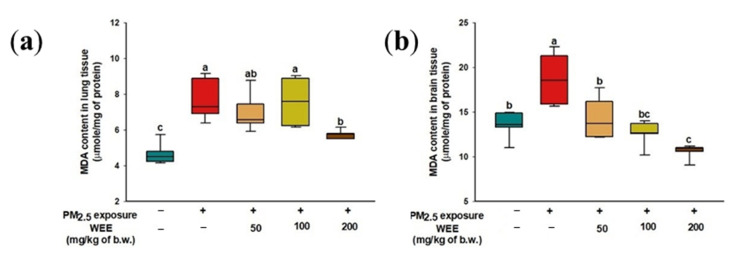
Inhibitory effect of water extract from *Ecklonia cava* (WEE) on fine dust (PM_2.5_)-induced lipid peroxidation in lung (**a**) and brain (**b**) tissues. Bars in box plot are colored by group (green-colored bars; normal control (NC), red-colored bars; PM_2.5_ exposure (PM_2.5_), yellow-colored bars; WEE 50, light green-colored bars; WEE 100, brown-colored bars; WEE 200). The results are shown with a mean of ± SD (*n* = 7), and they were considered statistically significant at *p* < 0.05. Different small letters ^(a–c)^ indicate a statistical difference.

The PM_2.5_-exposed mice (7.66 μmole/mg of protein) had lipid peroxidation in the lung tissue compared to the NC group (4.65 μmole/mg of protein), while the WEE 200 group (5.75 μmole/mg of protein) showed a significant decrease in MDA content compared to the WEE 50 and 100 groups (6.79 and 7.59 μmole/mg of protein, respectively) (Figure 3a). 

In brain tissue, the MDA content was higher in the PM_2.5_-exposed group (18.71 μmole/mg of protein) than in the NC group (13.56 μmole/mg of protein) (Figure 3b). In contrast to the PM_2.5_-exposed group, the WEE 200 group (10.62 μmole/mg of protein) exhibited a significant decrease in MDA content.

### 2.4. Mitochondrial Function

#### 2.4.1. Mitochondrial Function in Lung Tissue

**Figure 4:**
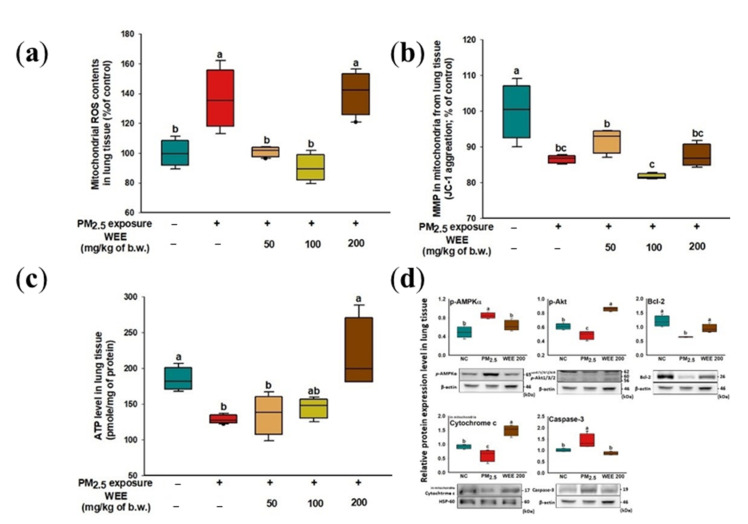
Effect of water extract from *Ecklonia cava* (WEE) on fine dust (PM_2.5_)-induced mitochondrial dysfunction in lung tissue. Mitochondrial ROS content (**a**), mitochondria membrane potential (MMP) (**b**), ATP levels (**c**), band images of Western blot analysis and the expression level of mitochondria-related apoptosis signaling molecules (**d**) on PM_2.5_-induced mitochondria damage in lung tissue. Bars in box plot are colored by group (green-colored bars; normal control (NC), red-colored bars; PM_2.5_ exposure (PM_2.5_), yellow-colored bars; WEE 50, light green-colored bars; WEE 100, brown-colored bars; WEE 200). The results are shown with a mean of ± SD (*n* = 5), and were considered statistically significant at *p* < 0.05. Different small letters ^(a–c)^ indicate a statistical difference.

The mitochondrial ROS content increased from the PM_2.5_-exposed group (136.34%) compared to the NC group (Figure 4a). The WEE 50 and 100 groups (101.00% and 89.95%, respectively) showed a significant decrease in mitochondrial ROS content in the lung tissue, while the WEE 200 group (140.45%) showed no significant differences. The PM_2.5_-exposed group (86.59%) showed a decreased MMP compared to the NC group (100.00%) in Figure 4b. The administration of the WEE had no statistical difference in MMP with the PM_2.5_-exposed group. In Figure 4c, the PM_2.5_-exposed mice (128.57 pmole/mg of protein) showed about a 31.92% decrease in ATP levels compared to the NC group (184.45 pmole/mg of protein). The WEE 200 group (217.22 pmole/mg of protein) showed a significant increase in ATP level compared with the other WEE groups (WEE 50: 135.75, WEE 100: 145.02 pmole/mg of protein). The proteins of the mitochondria-related apoptosis mechanism (*p*-AMPKα, *p*-Akt, Bcl-2, mitochondrial cytochrome C, and cleaved caspase-3) were analyzed using a Western blot assay (Figure 4d). The decreased ATP content by PM_2.5_ led to an increase in the AMP/ATP ratio, and as a result, the *p*-AMPKα expression increased. In the PM_2.5_-exposed group, the *p*-AMPKα expression led to a decrease in *p*-Akt and Bcl-2 expression. The cleaved caspase-3 expression increased with PM_2.5_ exposure, leading to mitochondrial cytochrome C being released into the cytosol. The WEE 200 group effectively regulated the mitochondria-related apoptosis molecules in lung tissue.

#### 2.4.2. Mitochondrial Function in Brain Tissue

**Figure 5:**
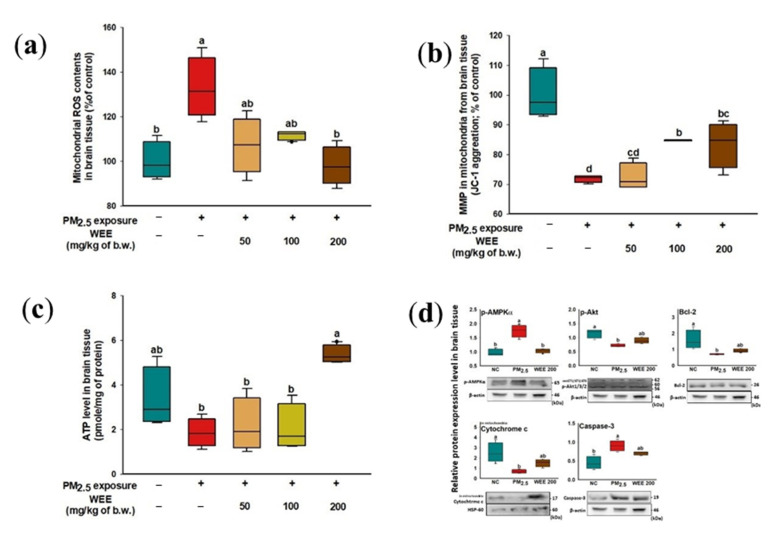
Effect of water extract from *Ecklonia cava* (WEE) on fine dust (PM_2.5_)-induced mitochondrial dysfunction in brain tissue. Mitochondrial ROS content (**a**), MMP (**b**), ATP levels (**c**), and band images of Western blot analysis and the expression level of mitochondria-related apoptosis signaling molecules (**d**) on PM_2.5_-induced mitochondria damage in brain tissue. Bars in box plot are colored by group (green-colored bars; normal control (NC), red-colored bars; PM_2.5_ exposure (PM_2.5_), yellow-colored bars; WEE 50, light green-colored bars; WEE 100, brown-colored bars; WEE 200). The results are shown with a mean of ± SD (*n* = 5), and were considered statistically significant at *p* < 0.05. Different small letters ^(a–d)^ indicate a statistical difference.

In brain tissue, the increase in mitochondrial ROS content was caused by PM_2.5_ exposure (132.83%) compared to the NC group (100.00%) (Figure 5a). On the other hand, the administration of the WEE 200 group (98.10%) more effectively inhibited the production of ROS than in the WEE 50 and 100 groups (107.09% and 111.63%, respectively). As shown in Figure 5b, the PM_2.5_-exposed group (71.82%) exhibited a lower MMP than the NC group (100.00%). In comparison to the PM_2.5_-exposed group, the MMP of the WEE groups (WEE 50: 72.29%, WEE 100: 84.74%, and WEE 200: 83.47%) improved. The PM_2.5_-exposed group (1.86 pmole/mg of protein, about a 44.48% decrease) indicated decreased ATP levels in the brain tissue compared to the NC group (3.35 mole/mg of protein) (Figure 5c). On the other hand, the WEE 200 group (5.36 mole/mg of protein) showed an increase in ATP level compared to the PM_2.5_-exposed group. Similar to the results of testing the lung tissue (Figure 4), *p*-AMPKα was activated by PM_2.5_ exposure, and *p*-Akt and Bcl-2 showed a decreased expression level compared to the NC group in the brain tissue (Figure 5d). The WEE 200 group effectively regulated the *p*-AMPKα, *p*-Akt, and Bcl-2. Then, the release of cytochrome C into the cytosol led to caspase-3 activation. These results suggest that the administration of the WEE 200 effectively inhibited the mitochondrial damage and the mitochondria-related apoptosis from PM_2.5_ exposure in brain tissue.

### 2.5. Cognitive Function-Mediated Molecule Analysis

#### 2.5.1. Aβ Production/tau Hyperphosphorylation Signaling Pathways

**Figure 6:**
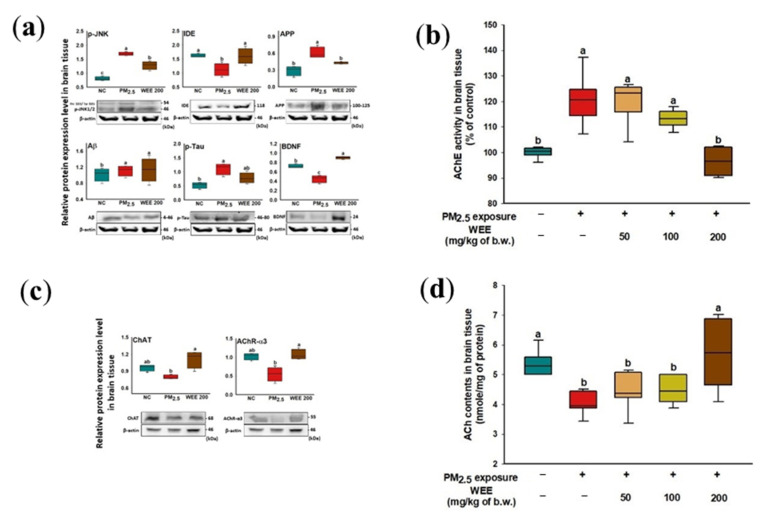
Effect of water extract from *Ecklonia cava* (WEE) on fine dust (PM_2.5_)-induced cognitive dysfunction. Band images of Western blot analysis and the expression level of Aβ production/tau hyperphosphorylation-mediated molecules (**a**), AChE activity (**b**), band images of Western blot analysis for cholinergic activity and the expression level of cholinergic system signaling molecules (**c**) and ACh contents (**d**) in brain tissue. Bars in box plot are colored by group (green-colored bars; normal control (NC), red-colored bars; PM_2.5_ exposure (PM_2.5_), yellow-colored bars; WEE 50, light green-colored bars; WEE 100, brown-colored bars; WEE 200). The results are shown with a mean of ± SD (*n* = 5), and were considered statistically significant at *p* < 0.05. Different small letters ^(a–c)^ indicate a statistical difference.

The increase in *p*-JNK as the result of oxidative stress by PM_2.5_ exposure was detected and induced the decrease in IDE expression, which is an amyloid-beta degradation enzyme (Figure 6a). However, there was no significant increase in Aβ, but an increase in APP expression was detected. *p*-tau expression increased, and a significant decrease in BDNF expression was indicated. The administration of the WEE promoted a decrease in APP and *p*-tau via inhibition of *p*-JNK and indicated a significant increase in BDNF as neurotrophic factor.

#### 2.5.2. Cholinergic Function

In Figure 6b, AChE activity increased approximately 120.51% compared to the control group (100.00%) in the brain tissue on PM_2.5_-induced cholinergic system impairment mice. The administration of the WEE (WEE 200: 96.43%) significantly prevented PM_2.5_-induced AChE activation, similar to that of the control group. The WEE 200 group also showed a significant increase in ChAT and AChR-α3 expression compared with the PM_2.5_ exposure group (Figure 6c). Finally, the ACh content in the PM_2.5_-exposed group (4.07 nmole/mg of protein; 24.49% decrease) decreased compared to the NC group (5.39 nmole/mg of protein) (Figure 6d). On the other hand, the WEE 200 group (5.67 nmole/mg of protein) had effectively increased ACh content in the mouse brain tissue compared with the PM_2.5_-exposed group by regulating AChE activity, ChAT, and AChR-α3 expression.

### 2.6. Main Compound Analysis

#### 2.6.1. Chemical Composition of Polysaccharide

The total polysaccharide content exhibited was 34.26% in the WEE, and their average molecular weight was analyzed to be 160.13 kDa (Table 1). The sulfate content was 17.03%, and monosaccharides were composed as follows: fucose (9.76%), rhamnose (16.03%), galactose (6.53%), glucose (6.65%), xylose (48.97%), and other monosaccharides (12.06%).

#### 2.6.2. Phenolic Compounds Analysis

**Figure 7:**
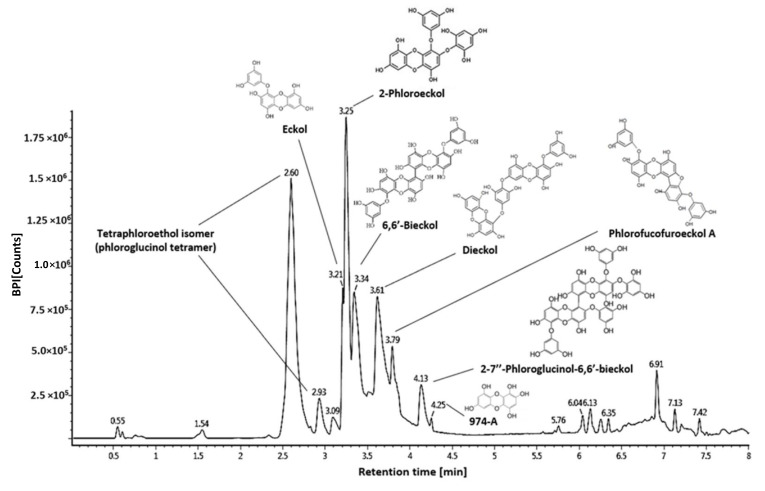
Analysis of major phenolic compounds of *Ecklonia cava* using the UPLC-Q-TOF system. MS chromatogram and chemical structure of the identified compound.

The phenolic compounds such as physiological compounds in the WEE were identified using the UPLC-QTOF MS system, and proposed compounds were identified by comparison with LC-MS/MS fragments from previous literature (Figure 7, Table 2). Tetraphloroetiol isomer (phloroglucinol tetramer) was detected at 2.60 min (*m*/*z* 497.11278: 327, 265, 231, 139) and 2.93 min (*m*/*z* 497.11278: 371, 353, 231, 229, 138, 125) [15]. Eckol (*m*/*z* 371.04088: 353, 263, 245, 201 at 3.21 min), 2′-phloroeckol (*m*/*z* 495.09875: 477, 387, 263, 244, 231, 229, 201 at 3.25 min), 6′6′-bieckol (*m*/*z* 741.13508: 723, 490, 477, 244, 229, 201 at 3.34 min), dieckol (*m*/*z* 741.13508: 615, 493, 491, 477, 369, 261, 229, 201 at 3.62 min), phlorofuroeckol A (*m*/*z* 601.11104: 493, 492, 385, 366, 244, 299 at 3.79 min), 2,7″-phloroglucinol 6,6′-bieckol (PHB) (*m*/*z* 973.19218: 741, 602, 601, 493, 370, 229 at 4.13 min,), and 974-A (*m*/*z* 973.19218: 829, 707 493, 479, 353, 335, 229 at 4.25 min) were identified as major compounds [16,17].

## 3. Discussion

PM_2.5_ exposure into central airways is known to cause pulmonary dysfunction, and these reactions also induce systemic oxidative damage and an inflammatory reaction [18]. Among these types of damage, PM_2.5_ exposure is associated with neurodegeneration such as AD, PD, multiple sclerosis, and amyotrophic lateral sclerosis [19]. In particular, the inhaled PMs could be translocated through various routes (e.g., epithelial barriers, olfactory, and sensory neuronal routes) into secondary organs including the brain beyond the respiratory system [19]. In our results (Figure 1), the PM_2.5_-exposed mice indicated learning and memory impairments, and the intake of the WEE prevented cognitive dysfunction. According to Zhang et al. (2018) [20], PM_2.5_ containing metallic elements and PAHs were intratracheally injected into Sprague–Dawley male rats, and the results of the open-field and Morris water maze tests indicated spatial learning and memory impairments. These results were reported to be due to the changes in mitochondria and myelin sheaths by increasing the expression of apoptosis-related proteins such as caspase-3/9 in the brain tissue. Research on the mechanism of cognitive dysfunction is still insufficient. However, PM_2.5_ has been reported to be highly correlated with cognitive function or dementia among the many various pollutants (CO, NO_2_/NO_X_, O_3_, PM_2.5_, and PM_10_) [21].

The toxicity response of PMs is posited as a three-step model: the defense mechanism at a low level of oxidative stress (step 1), inflammatory response (step 2), and toxicity (step 3) [22]. Therefore, the inflammatory response is an important factor in PM-induced organ toxicity and dysfunction. In particular, the regulation of the inflammatory response in the lungs is an important mechanism in PM-related studies because PMs are absorbed through the respiratory tract [5]. The pro-inflammatory response is mediated by MAPK and NF-κB cascades, and it induces cytokine production. The pro-inflammatory cytokine production by PMs participates in local and systemic inflammatory response through the blood. Furthermore, PM_2.5_ reaching the alveoli can translocate into the blood through the blood–air barrier, and this affects other organ systems [6]. Among the other organs, inflammation in the brain can be triggered through the translocation of PMs from the blood and olfactory bulb. In addition, pro-inflammatory cytokines production via olfactory bulbs and lungs contributes to B-B-B damage, and it affects neurons in diffuse areas of the brain such as the frontal cortex and hippocampus, leading to an inflammatory response. TLRs are present on gastrointestinal epithelial cells, macrophages, neurons of the enteric nervous system, primary afferent neurons, and various brain cells (neurons, microglial cells, and astrocytes) [23]. 

TLRs and NLR signaling mechanisms are known to be important for PM-mediated immune response, and these receptors activated NF-κB by breaking the combination of NF-κB/IκB through phosphorylation of IκB (degradation) [24,25]. In some studies, inflammation by PM_2.5_ exposure has been associated with the formation of the nucleotide-binding oligomerization domain-like receptor (NLR) family, pyrin domain-containing 3 (NLRP3) inflammasome. Moreover, formatted NLRP3 inflammasome activates the pro-inflammatory cytokines (IL-18 and IL-1β) through caspase-1 activation [26]. As the lung–brain axis, pulmonary inflammation affects cognitive function, and recent research reported that lung damage influences brain function [27]. The production of pro-inflammatory cytokines in the lungs leads to systemic inflammation, and it triggers the disruption of the B-B-B tight-junction. The B-B-B increases permeability, resulting in cognitive deficiencies. Therefore, the regulation of the inflammatory reaction in the lungs plays an important role in improving cognitive function. Some studies have been reported to focus on the effect of fucoidan from brown seaweed on inflammation. The fucoidan isolated from *Padina commersonii* downregulated the NF-κB signaling pathway by inhibiting the TLR-2/4 and myeloid differentiation primary response 88 (MyD 88) in LPS-stimulated RAW 264.7 cells [28]. In addition, the fucoidan extract from brown seaweeds exhibited an anti-inflammatory effect by inhibiting nitric oxide (NO) production and prostaglandin E_2_, NO synthase, cyclooxygenase-2, monocyte chemoattractant protein-1, and pro-inflammatory cytokines (IL-1β, TNF-α) through the down-regulation of the NF-κB/MAPK/AKT pathway in LPS-stimulated BV-2 microglia cells [29]. Therefore, this suggests that the anti-inflammatory effect in the lungs of the WEE, as well as in the brains, could have helped to improve cognitive function (Figure 2). 

PM_2.5_ as a systemic toxicity agent causes a pro-oxidant/antioxidant imbalance or free radical overproduction in multiple organs, and the result leads to a decrease in glutathione levels and superoxide dismutase, catalase, and glutathione peroxidase activities [1]. As a result, PM_2.5_-exposed mice had a significant increase in lipid peroxidation in various organs such as the heart, liver, spleen, lungs, kidneys, brain, and testicles. The lungs play an essential role in mediating blood circulation and extrapulmonary organs [1]. However, the lungs are very vulnerable to PM-induced oxidative damage due to being directly affected by air pollutants. 

The cerebral tissue consuming the high oxygen for energy production leads to excessive mitochondrial ROS production and increases microglial ROS and cytokines in a perpetual process. The free radicals and their derivatives such as superoxide anions, hydrogen peroxide (H_2_O_2_), and hydroxyl radicals (^•^OH) cause neuronal injury and dysfunction, and oxidative stress causes the onset of neurological disorders (AD, PD, vascular dementia, anxiety, and depression) [30]. ROS could directly downregulate proteins of tight junctions and also indirectly activate matrix metalloproteinases damaging the B-B-B. In particular, brains that are rich in polyunsaturated fatty acids in the neuronal membrane are vulnerable to ROS [31]. Therefore, previous reports have suggested that some antioxidants can be used mechanistically and therapeutically for neurological disorders by attenuating major oxidative stress markers [32]. In addition, the inhibition of oxidative damage in the brain plays an important role in the prevention of neurodegenerative diseases that can occur as a result of other processes such as neuroinflammation and protein misfolding [19]. 

Mammalian mitochondria have been considered to be important organelles because they play a key role in energy production, cell metabolism, programmed cell death, calcium homeostasis, and other biochemical functions [33,34,35]. The correlation between PM_2.5_ and mitochondrial dysfunction is still unclear. However, some studies have found that PM_2.5_ causes damage to various organs through mitochondrial dysfunction. Mitochondria continuously undergo the fusion/fission process by maintaining their function. Breaking this balance leads to cell damage/death. In particular, neurons, which require high amounts of energy, contain thousands of mitochondria [9]. Inhibition of ATP production by mitochondrial damage activates *p*-AMPKα expression. And the excessive *p*-AMPKα expression causes the alterations in Bcl-2 family members such as Bcl-2 and BAX that are related to apoptosis [18]. A decrease in Bcl-2 and increase in BAX induces pore formation in the outer membrane of the mitochondria. The released cytochrome C through the pore is involved in apoptosome formation. Finally, cell apoptosis is caused by the activation of caspase through apoptosome formation. PMs cause apoptosis through caspase-3 activation by the intrinsic (mitochondrial) pathway, which triggers apoptosome formation [6]. In addition, pro-inflammatory response by PMs can affect organ toxicity by mitochondrial dysfunction and the apoptotic pathway [22]. Lee et al. (2019) [36] suggested the possibility that eckol, dieckol, and 8,8′-bieckol, which are the main biological compounds of *E. cava,* are new therapeutic agents for AD due to their anti-inflammatory and anti-apoptotic effects on Aβ_25-35_-induced cytotoxicity in PC-12 cells. In particular, dieckol and 8,8′-bieckol effectively regulated the mitochondria-related apoptotic proteins (caspase-8/9/3 and PARP-1) by inhibiting the intrinsic/extrinsic pathways; however, eckol only inhibited the intrinsic pathway by affecting the caspase-9, -3, and PARP-1. Moreover, in a traumatic brain injury model, fucoidan effectively restored mitochondrial function by decreasing the ROS overproduction and mitochondrial permeability transition pore opening and increasing the ATP synthesis through upregulation of sirtuin-3, which is located in the mitochondrial matrix as molecules for mitochondrial modulation [15]. As a result, fucoidan alleviates mitochondrial-initiated apoptosis and stimulates the activation of antioxidant molecules (Mn-SOD, GSH, and catalase) through regulation of gene expression of forkhead box O3 (FOXO3A) and nuclear erythroid 2-related factor 2 (Nrf-2)-antioxidant response element (ARE), which is an antioxidant response element-dependent gene that regulates the expression of antioxidant molecules. Notably, the oxidative damage in the brain plays an important role in the outbreak of neurodegenerative diseases as a result of other processes such as neuroinflammation and protein misfolding [37]. Therefore, the WEE proved to be a functional substance for PM_2.5_-induced oxidative damage through inhibition of lipid peroxidation and mitochondria activation (Figure 3, Figure 4 and Figure 5).

Based on the above results, it was confirmed that PM_2.5_ exposure caused learning and memory decline by inducing oxidative damage and neuroinflammation in the brain tissue. As a result, the PM_2.5_ exposure altered Aβ production/tau hyperphosphorylation and cholinergic molecules, and the intake of WEE effectively regulated the cognition-related proteins (Figure 6). The Aβ production/tau hyperphosphorylation is reported as being major hallmarks of cognitive decline [21]. Activated *p*-JNK by oxidative stress inhibited IDE expression as the Aβ peptide degrading enzyme, and the results induced Aβ production/aggregation. In addition, *p*-JNK caused the inhibition of the PI3K/Akt/GSK-3 signaling pathway as a cell growth- and survival-related mechanism, and the results induced tau hyperphosphorylation [38]. BDNF, a member of the neurotrophic factor family, is closely related to the protection of neuronal cells, synaptic plasticity by promoting neurogenesis, and memory formation in the brain [10]. BDNF can activate many kinds of pathways related to neural function by combining with the high-affinity tyrosine receptor kinase B (TrkB), and the result is a downregulated cyclic adenosine monophosphate response element-binding protein (CREB), which plays an important role in neuronal plasticity and long-term memory formation in the brain tissue [10]. Fucosterol from *Ecklonia stolonifera* enhanced the cognitive function and inhibited the soluble Aβ peptide by up-regulating the mature BDNF/TrkB and glucose-regulated protein-78 expression through the tyrosine receptor kinase B-mediated ERK1/2 activation and the JNK inhibition in the hippocampal neurons of aging mice [39].

Oxidative stress and inflammation in the brain trigger a cascade of synaptic (function) failures, synapse loss, and neuronal cell death, and eventually lead to dementia [40]. The cholinergic system has been considered as the target for the pathophysiology and treatment of AD, and the “cholinergic hypothesis” is important in brain function [41]. In brief, ACh,a crucial neurotransmitter, is synthesized by ChAT combined with acetyl-CoA synthesized from mitochondrial pyruvate in cholinergic neurons. Synthesized ACh is released into the synapse and combined with ACh receptors such as muscarinic or nicotinic receptors for signal transmission [42]. On the other hand, in the brains of AD patients, ACh breaks down into choline and acetate by AChE activation, and the results show a decrease in ACh content. Hence, ChAT activation and AChE inhibition are effective for cognitive improvement by keeping neurotransmission going through the regulation of ACh content. Phlorotannins, as major bio-active compounds of *E. cava,* showed high potential as AChE inhibitors [43]. The hydroxyl group of eckol, dieckol, and 8,8′-bieckol inhibited AChE activity by forming hydrogen bonds in the active site of AChE (Eckol; TRP86, THR83, TRP86, TYR124, and SER125, Dieckol; ASN233, THR238, ARG296, and HIS405, 8,8′-bieckol; ARG296). In recent years, several natural compounds have been found to have a kind of neuroprotective role or anti-amyloid activity. In particular, homotaurine has been reported as a therapeutic agent for AD patients. The major mechanism of homotaurine is the activation of cholinergic transmission (ACh release) through the regulation of γ-aminobutyric acid (GABA) A receptor affinity, and it interfered with the cascade of amyloidogenic reaction in mild cognitive impairment (MCI) individuals [44]. Similar to what was suggested in previous studies, *E. cava* could be used as a potential therapeutic and preventing agent for cognitive dysfunction symptoms with the cholinergic transmission activation and anti-amyloidogenic effect in MCI and AD patients. However, further properly designed clinical studies in animals and humans are needed however to fully demonstrate the action of *Ecklonia cava* in treatment of AD patients.

The fucoidan and phlorotannin were confirmed as the results of the major bioactive compounds analysis in WEE (Table 1 and Table 2 and Figure 7). The major polysaccharides of brown seaweed were laminarin, fucoidan, and alginate [14]. Among polysaccharides, the fucoidans, which are a class of fucose-rich sulfated homo-/heteropolysaccharides, are constituents of brown seaweed and some marine invertebrates. The fucoidans are reported to play a role in various physiological activities including anti-coagulant, anti-thrombotic, anti-inflammatory, and reducing blood lipids [45]. According to these reports, fucoidans have been investigated in recent years to develop treatment agents and functional foods. Among the various physiological effects of fucoidans, an anti-inflammatory effect is reported as the main physiological function. During the last 5-10 years, evidence on the anti-inflammatory effects of fucoidans has been found. Fucoidan effectively modulated the pro-inflammatory enzyme activity, inflammation-related gene expression, transcription factors, adhesion molecules, matrix metalloproteinases, and complement cascade properties [46]. Fucoidan from *E. cava* had strong anti-inflammatory effects on an LPS-induced zebrafish model through the inhibition of ROS and NO generation [47]. The fucoidans from *Turbinaria decurrens* exhibited anti-inflammatory activity by upregulation of the antioxidative system (superoxide dismutase, catalase, and glutathione peroxidase, glutathione-S-transferase, and glutathione activity) and the regulation of pro-inflammatory mediators (IL-1β, cyclooxygenase-2, matrix metalloproteinases-9, and NF-κB pathway) on formalin-induced paw-edema in mice [48].

The bioactivity of fucoidans plays an important role in their structures, and the high molar ratio of sulfate and their size determines their physiological activities. Fucoidan, composed of 100–1600 kDa, is known to be unable to pass through the B-B-B. This means that fucoidan cannot directly affect other organs [49], and intracellular transport is difficult, so it cannot move into the brain or other organs by simple diffusion. However, recent research reported the effects of fucoidan on brain function. The results suggest that fucoidan may be involved in cell influx by transporters. One recent hypothesis predicts that the absorption of fucoidan through the B-B-B into the brain could be influenced by the sodium-glucose transporter or glucose transporter-2, considering the affinity of polysaccharides [15]. In addition, the unabsorbed fucoidan in the digestive system can affect cognitive function through fermentation by the intestinal microbiota such as the *Lactobacillus* strains in humans [49]. The function as prebiotics has been associated with brain function with either a direct or indirect effect on signaling molecules by producing gut hormones as a neuropeptide (e.g., peptide YY), hippocampal BDNF, and the *N*-methyl-d-aspartate receptor subunit-1.

Phlorotannins have been known as unique bioactive compounds in brown seaweed, and they were formed by the polymerization of phloroglucinol (1,3,5-tryhydroxybezene) through the acetate-malonate pathway as a polyketide pathway [31,50]. Phlorotannins are oligomeric phenolic compounds containing eckol (trimer), 6.6′-bieckol (hexamer), dieckol (hexamer), and phlorofucofuroeckol (pentamer) with a wide range of molecular weights (126–65,000 Da) [50]. Small molecules that are less than 500 Da and can pass through the B-B-B and its tight junctions and exert these abilities of the B-B-B exert a stronger beneficial effect as therapeutic agents for neurodegenerative diseases [34,51]. Among phlorotannins, phloroglucinol and eckol can directly affect cognitive function because they can pass through the B-B-B, and phloroglucinol is detected in the brain tissue [34,52]. Aβ, which is a major pathogenic peptide in AD, is formatted by the enzymatic cleavage of APP by β- and γ-secretase, which is known as amyloidogenic processing [53]. However, activation of α- and γ-secretase induces the non-amyloidogenic processing of APP, and inhibited Aβ production. The phlorotannin-rich extract from *E. cava* inhibited the mechanism of Aβ formation by the activation of α-secretase in HEK293 APP Swedish stable cells [53]. Phloroglucinol is a component of phlorotannins that effectively attenuates the cognitive decline in the 5Xfamilial AD (FAD) mouse model. These results were observed to restore the dendritic spine density in the CA1 region of the hippocampus and synaptic proteins such as synaptophysin and postsynaptic density protein-95 and reduce the ionized calcium-binding adapter molecule 1, glial fibrillary acidic protein, and mRNA levels of pro-inflammatory cytokines (TNF-α and IL-6) [54]. Phlorotannin-rich extract from *Ishige foliacea* improved the cognitive function by upregulating the ERK/CREB/BDNF signaling pathway and preventing the AChE activity and oxidative stress on the scopolamine-induced learning and memory impairment mouse model [55].

## 4. Materials and Methods

### 4.1. Chemicals

Acetylthiocholine, thiobarbituric acid, trichloroacetic acid, phosphoric acid, digitonin, and all other chemicals used were purchased from Sigma-Aldrich Chemical Co. (St. Louis, MO, USA). PM_2.5_ was purchased from Powder Technology Inc. (Arizona Test Dust, Arden Hills, MN, USA). In brief, this dust that accumulated in the air from around tractors or appliances was collected from the Salt River Valley, Arizona and then fractionated into sizes of 0-3 μm. Anti-AChRα3 (sc-365479), anti-p-IκB-α (sc-8404), anti-Bcl-2 (sc-509), anti-caspase-1 (sc-392736), anti-β-amyloid (sc-28365), anti-p-Tau (sc-12952), anti-cytochrome c (sc-13560), anti-p-Akt1/2/3 (sc-101629), anti-p-JNK (sc-6254), anti-IDE (sc-393887), anti-TLR-4 (sc-52962), and anti-β-actin (sc-69879) were purchased from Santa Cruz Biotechnology (Santa Cruz, CA, USA), and anti-IL-1β (sc-12742), anti-NF-κBp65 (#6956), anti-p-AMPKα (#2531), and secondary antibodies were purchased from Cell Signaling Technology (Danvers, MA, USA). Anti-ChAT (20747-1AP) was purchased from Proteintech (Wuhan, China), and anti-BDNF (CSB-PA05775A0Rb) and anti-caspase-3 (CSB-PA05689A0Rb) were purchased from Cusabio Biotech (Wuhan, China).

### 4.2. Sample Preparation

*E. cava* was obtained from Parajeju (Jeju, Korea) in February 2018. The *E. cava* was washed and lyophilized using a vacuum-tray freeze dryer (Operon, Gimpo, Korea). Powdered *E. cava* was extracted 50 times with volumes of distilled water at 40 °C for 2 h, and was filtered through Whatman No. 2 filter paper (Whatman International Limited, Kent, UK). Water extract from *E. cava* was concentrated using a rotary vacuum evaporator (N-1000; EYELA Co., Tokyo, Japan), lyophilized, and stored at −20 °C.

### 4.3. In Vivo Experimental Design

#### 4.3.1. Animals

BALB/c mice (male, 6 weeks) were obtained from Samtako (Osan, Korea), and mice were randomly assigned to four per cage (a 12 h light/dark cycle, 55% humidity, and 22 ± 2 °C). Each group (*n* = 14) was divided into 6 groups, and each consisted of a normal control (NC) group, a PM_2.5_-exposed group (negative control group; PM_2.5_), and WEE groups (50, 100, and 200 mg/kg of body weight, respectively; WEE 50, WEE 100, and WEE 200). The WEE was dissolved into drinking water and was orally fed using a stomach tube once a day for 12 weeks. WHO Air quality guidelines for fine particulate matter recommend expositions to PM_2.5_ < 25 μg/m^3^ 24 h mean in human, and the annual PM_2.5_ concentration in the most polluted cities in the world is around 170 μg/m^3^ [56,57]. The mice were exposed to PM_2.5_ at a 500 μg/m^3^ concentration in the whole-body exposure chamber (5 h/day and 5 days/week for 12 weeks).

#### 4.3.2. Y-Maze Test

The Y-maze consisted of three arms made of a black acrylic plate (33 cm long, 15 cm high, and 10 cm wide). Each mouse (*n* = 9 per groups) was located at the end of the arm, and then its movement was recorded using a video tracking system (Smart v3.0, Panlab, Barcelona, Spain) for 8 min.

#### 4.3.3. Passive Avoidance Test

The passive avoidance test box consisted of dark and light chambers. The mice (*n* = 9 per group) were placed in the light chamber for 2 min and then received an electronic shock (0.5 mA, 1 s) when it stepped into the dark chamber. After 24 h, the mice were placed again in the light chamber and the step-through latency time was measured when they re-entered the dark chamber (maximum time limit: 300 s).

#### 4.3.4. Morris Water Maze Test

A stainless-steel circular pool (150 cm in diameter and 60 cm in height) was separated into quadrants (N, S, E, and W zones), which were filled with melted squid ink (Cebesa, Valencia, Spain). In the center of the W zone, a hidden platform was located during the training periods. The mice (*n* = 9 per groups) were allowed to swim freely, and the escape latency time to the platform was recorded using a video tracking system (maximum time: 60 s). Training trials were repeated four times a day for four days. Lastly, a probe trial was conducted without the platform for 90 s, and the time spent in the W zone was recorded [58].

### 4.4. Biochemicals Analysis

#### 4.4.1. Preparation of the Tissue

After the behavioral test, the mice fasted for 8 h and were then sacrificed. The blood was drawn from the abdominal aorta. The lung and brain tissues were immediately collected for biochemical analysis and washed with ice-cold phosphate-buffered saline (PBS, pH 7.4) (137 mM NaCl, 2.7 mM KCl, 4.3 mM Na_2_HPO_3_, and 1.4 mM KH_2_PO_4_) and kept at −80 °C until used. The protein level was determined based on the Bradford method using Bio-Rad protein assay dye reagent (Bio-Rad Laboratories Inc., Hercules, CA, USA) [59].

#### 4.4.2. Western Blot Assay

The entire lung and brain tissues (*n* = 5 per group) were minced with a surgical scissor, and the small pieces were homogenized using a bullet blender (Next Advance Inc., Averill Park, NY, USA) with a lysis buffer containing 1% protease inhibitor. Afterward, these homogenates were immediately centrifuged at 13,000× *g* at 4 °C for 10 min. The proteins were electrophoresed on sodium dodecyl sulfate polyacrylamide gel and then transferred to a polyvinylidene difluoride membrane (Millipore, Billerica, MA, USA). The membranes were blocked with 5% skim milk and then incubated with the primary antibodies overnight at 4 °C. After incubation, the membranes were then reacted with the secondary antibodies for 1 h. The band images were detected with a Chemi-doc (iBright Imager, Thermo-Fisher Scientific, Waltham, MA, USA).

#### 4.4.3. Cytokine Contents

The small pieces of lung and brain tissue (*n* = 5 per groups) were homogenized using a 0.1% Tween 20 in PBS containing 1% protease inhibitor, and cytokines were analyzed using a mouse magnetic luminex assays kit (LXSAMSM-4) by MAGPIX^®^ instrument (Luminex Corporation, Austin, TX, USA), which utilizes xMAP technology and xPONENT 4.2 software (Luminex Corporation, Austin, TX, USA).

#### 4.4.4. MDA Content

The small pieces of lung and brain tissue (*n* = 7 per group) were homogenized with 10 volumes of ice-cold PBS using a bullet blender (Next Advance Inc., Averill Park, NY, USA). The homogenized tissues were centrifuged at 6000× *g* for 10 min. The supernatants were mixed with 1% phosphoric acid and 0.67% 2-thiobarbituric acid and then reacted at 95 °C for 1 h. After cooling, the mixture was measured at 532 nm using a spectrophotometer (UV-1201; Shimadzu, Kyoto, Japan).

### 4.5. Mitochondrial Activity

#### 4.5.1. Isolation of Mitochondria

To separate the lung and brain mitochondria, lung and brain tissue (*n* = 5 per groups) was added to the isolation buffer (215 mM mannitol, 75 mM sucrose, 0.1% BSA, 20 mM HEPES (Na^+^) and 1 mM ethylene glycol-bis(2-aminoethylether)-N,N,N′,N′-tetraacetic acid (EGTA)) and homogenated using a bullet blender (Next Advance Inc). The homogenate was spun down at 1300× *g* for 5 min, and the supernatant was centrifuged again at 13,000× *g* for 10 min. The pellet was then mixed with the isolation buffer containing 0.1% digitonin and left on ice for 5 min. After that, the mixture was added to the isolation buffer and centrifuged at 13,000× *g* for 15 min. The pellet was re-mixed with the isolation buffer without 1 mM EGTA and centrifuged at 10,000× *g* for 10 min [60].

#### 4.5.2. Mitochondrial ROS Content

To assess the ROS content of the mitochondria, the separated mitochondria (protein concentration: 0.8 mg/mL) were mixed with 25 μM DCF-DA in respiration buffer (125 mM KCl, 2 mM KH_2_PO_4_, 2.5 mM malate, 20 mM HEPES, 1 mM MgCl_2_, 5 mM pyruvate, and 500 μM EGTA) and left in the darkroom for 20 min. After incubation, the fluorescent intensity was measured (excitation wave: 485 nm, emission wave: 535 nm, Infinite F200, Tecan, Männedorf, Switzerland).

#### 4.5.3. Measurement of MMP

The tetraethylbenzimidazolylcarbocyanine iodide (JC-1) dye was added to the isolated mitochondria (protein concentration: 1.2 mg/mL) from the brain tissue in black 96 well and incubated for 20 min with aluminum foil. The fluorescence was measured to excitation wave (535 nm) and emission wave (590 nm).

#### 4.5.4. ATP Level

The ATP was extracted with 1% trichloroacetic acid (TCA) and 25 mM tris-acetate buffer (pH 7.8). The extract was centrifuged at 10,000× *g* for 15 min, and the supernatant was used. The ATP level was measured using a commercial kit (Promega, Madison, WI, USA) with a luminescence meter (Promega, Madison, WI, USA).

### 4.6. Measurement of Cholinergic Function

#### 4.6.1. AChE Activity

The brain homogenates (*n* = 7 per groups) with PBS buffer were directly centrifuged at 14,000× *g* for 30 min at 4 °C. The supernatant (2.5 mg/mL proteins) was mixed with 50 mM sodium phosphate buffer (pH 8.0) and then incubated for 15 min. After incubation, the mixture was added to 500 μM substrate solution (500 μM acetylthiocholine and 1 mM DTNB in a 50 mM sodium phosphate buffer) solution and reacted for 10 min. Then, the absorbance was measured at 405 nm using a microplate reader (EPOCH2; BioTek, Winooski, VT, USA) [61].

#### 4.6.2. ACh Contents

The supernatant was mixed with alkaline hydroxylamine reagent (2 M hydroxylamine in HCl and 3.5 N NaOH) and reacted at room temperature for 1 min (*n* = 7 per groups). The mixture was added to 0.5 N HCl and 0.37 M FeCl_3_ in 0.1 N HCl and measured at 540 nm [62].

### 4.7. Major Components Analysis

#### 4.7.1. Determination of Total Polysaccharide Contents

The total polysaccharide content was measured with a phenol-sulphuric acid method. A sample was mixed with a 5% phenol solution and sulphuric acid, and the mixture was shaken for 30 min. The absorbance was measured at 490 nm using a microplate reader (EPOCH2; BioTek, Winooski, VT, USA). The polysaccharide content was calculated based on the standard curve of glucose.

#### 4.7.2. Determination of Average Molecular Weight

The average molecular weight was evaluated using gel permeation chromatography (HLC-8320, Tosoh Bioscience, Stuttgart, Germany) with Tskgel guard PWxl+2xTSKgel GMPWxl+TSKgel G2500PWxl columns (7.8 × 300 mm, Tosoh Bioscience, Stuttgart, Germany) and equilibrated with 0.1 M NaNO_3_ for 60 min.

#### 4.7.3. Determination of Sulfate

The WEE was pretreated using 1 M HCl at 105 °C for 5 h in a water bath. The pretreated sample was added to 3% TCA and BaCl_2_-gelatin solutions and incubated for 15 min. The released barium sulfate suspension was measured at 360 nm using a microplate reader (EPOCH2; BioTek, Winooski, VT, USA). The sulfate content was calculated with the standard curve of potassium sulfate [63].

#### 4.7.4. Monosaccharide Composition

The monosaccharide composition of fucoidan was determined using a high-pH anion-exchange chromatography with pulsed amperometric detection (HPAEC-PAD) system (Dionex, Sunnyvale, CA, USA) using a CarboPacTM PA1 column (0.4 × 25 cm, Dionex, Sunnyvale, CA, USA) with 18 mM and 200 mM NaOH for 15 min.

#### 4.7.5. Major Phenolic Compound Analysis

For the analysis of the major phenolic compounds, we used Waters ACQUITY UPLC-Q-TOF/MS and a 2.1 × 100 mm × 1.7 μm ACQUITY UPLC BEH C18 column (Waters Corp, Milford, MA, USA) with a flow rate of 0.35 mL/min and oven temperature of 40 °C in negative ion mode. The solvent gradient of mobile phase A: distilled water containing 0.1% formic acid and B: acetonitrile containing 0.1% formic acid was used (0 to 2.0 min, 0.1% to 25% B, 2.0 to 8.0 min, 25% to 50% B). The conditions for MS analyses were applied to the drying gas (N_2_) temperature at 120 °C, drying gas flow at 30 L/h, ramp collision energy at 20–40 V, nebulizer pressure at 40 psi, fragmented voltage at 175 V, capillary voltage at 3 kV, and mass range from *m*/*z* 100 to 1500.

### 4.8. Statistical Analysis 

All data are expressed as the mean ± SD. The statistical significance of differences among groups was calculated by a one-way analysis of variance (ANOVA). Significant differences were determined using Duncan’s new multiple-range test (*p* < 0.05) of SAS ver. 9.4 (SAS Institute Inc., Cary, NC, USA). 

## 5. Conclusions

This study tried to evaluate the preventing effect of *E. cava* on PM_2.5_-induced cognitive dysfunction. The intake of WEE prevented the learning and memory impairment by PM_2.5_. The WEE groups showed an anti-inflammatory effect by regulating the inflammasome and pro-inflammatory cytokine production on TLR-4-initiated pro-inflammatory response in the lung and brain tissues. The WEE activated the mitochondrial function and effectively regulated mitochondria-mediated apoptosis molecules. Notably, the WEE group effectively regulated the cognition-related signaling pathway (Aβ production/tau hyperphosphorylation and cholinergic system) in brain tissue. The bioactive compound of *E. cava* was detected as fucoidans and phlorotannins. Therefore, these findings suggest that *E. cava* could be used as a potential material for preventing cognitive dysfunction through the regulation of inflammation, the mitochondria-mediated apoptosis pathway, and cognition-related molecules on PM_2.5_-induced cognitive dysfunction.

## Figures and Tables

**Table 1:** Molecular weight and composition (*w/w*% of dried weight) of water extract from *Ecklonia cava* (WEE).

Total Polysaccharide (%)	Average Molecular Weight (kDa)	Sulfate (%)	Relative Area (%)					
			Fucose	Rhamnose	Galactose	Glucose	Xylose	Others
34.26	160.13	17.03	9.76	16.03	6.53	6.65	48.97	12.06

**Table 2:** LC-MS/MS fragments of the identified compounds.

No.	RT (min)	*m*/*z* [M − H]^−^	LC-MS/MS Fragments	Proposed Compounds
1	2.60	497.11278	327, 265, 231, 139	isometric tetramer (phlorotannin oligomer)
2	2.93	497.11278	371, 353, 231, 229, 138, 125	isometric tetramer (phlorotannin oligomer)
3	3.21	371.04088	353, 263, 245, 201	Eckol
4	3.25	495.09875	477, 387, 263, 244,231,229,201	2-Phloroeckol
5	3.34	741.13508	723, 490, 477, 244, 229, 201	6′6′-Bieckol
6	3.62	741.13508	615, 493, 491, 477, 369, 261, 229, 201	Dieckol
7	3.79	601.11104	493, 492, 385, 366, 244, 299	Phlorofuroeckol A
8	4.13	973.19218	741, 602, 601, 493, 370, 229	2,7″-Phloroglucinol 6,6′-bieckol (PHB)
9	4.25	973.19218	829, 707 493, 479, 353, 335, 229	974-A
10	6.91	642.4498	363, 362, 279, 99, 85	Unknown
